# Whole Genome Expression Profiling of Semitendinosus Tendons from Children with Diplegic and Tetraplegic Cerebral Palsy

**DOI:** 10.3390/biomedicines11112918

**Published:** 2023-10-28

**Authors:** Simona Nemska, Simone Serio, Veronica Larcher, Giulia Beltrame, Nicola Marcello Portinaro, Marie-Louise Bang

**Affiliations:** 1 Milan Unit, Institute of Genetic and Biomedical Research (IRGB), National Research Council (CNR), 20138 Milan, Italy; simona.nemska@humanitasresearch.it (S.N.); simone.serio@humanitasresearch.it (S.S.); 2 IRCCS Humanitas Research Hospital, Rozzano, 20089 Milan, Italy; 3 Institute of Cardiovascular Regeneration, Goethe University, 60590 Frankfurt, Germany; larcher@med.uni-frankfurt.de; 4 Residency Program in Orthopedics and Traumatology, University of Milan, 20100 Milan, Italy; beltramegiulia.gb@gmail.com; 5 Department of Clinical Sciences and Community Health, University of Milan, 20122 Milan, Italy; nicola.portinaro@unimi.it; 6 Department of Pediatric Surgery, Fondazione IRCCS Ca’ Granda Ospedale Maggiore Policlinico, 20100 Milan, Italy

**Keywords:** cerebral palsy, tendons, extracellular matrix, gene expression, RNA-sequencing

## Abstract

Cerebral palsy (CP) is the most common movement disorder in children, with a prevalence ranging from 1.5 to 4 per 1000 live births. CP is caused by a non-progressive lesion of the developing brain, leading to progressive alterations of the musculoskeletal system, including spasticity, often leading to the development of fixed contractures, necessitating tendon lengthening surgery. Total RNA-sequencing analysis was performed on semitendinosus tendons from diplegic and tetraplegic CP patients subjected to tendon lengthening surgery compared to control patients undergoing anterior cruciate ligament reconstructive surgery. Tetraplegic CP patients showed increased expression of genes implicated in collagen synthesis and extracellular matrix (ECM) turnover, while only minor changes were observed in diplegic CP patients. In addition, tendons from tetraplegic CP patients showed an enrichment for upregulated genes involved in vesicle-mediated transport and downregulated genes involved in cytokine and apoptotic signaling. Overall, our results indicate increased ECM turnover with increased net synthesis of collagen in tetraplegic CP patients without activation of inflammatory and apoptotic pathways, similar to observations in athletes where ECM remodeling results in increased tendon stiffness and tensile strength. Nevertheless, the resulting increased tendon stiffness is an important issue in clinical practice, where surgery is often required to restore joint mobility.

## 1. Introduction

Tendons are fibrous connective tissues that connect skeletal muscle to bone and transmit the mechanical force generated by muscle contraction to the bones, driving the movement of the skeletal system. Tendons are composed primarily of cells and extracellular matrix (ECM), principally comprising collagen type I, which accounts for about 65–80% of the dry weight and is organized into parallel fiber bundles arranged into a three-dimensional network surrounded by a thin connective tissue sheath known as the epitenon [1]. Additionally, the ECM contains proteoglycans (e.g., aggrecan, versican, decorin, biglycan, fibromodulin, lumican, keratocan, osteoglycin, and syndecans), glycoproteins (e.g., elastin, cartilage oligomeric matrix protein (COMP/TSP5), tenascin-C, lubricin, tenomodulin, and fibronectin), glycosaminoglycans, and smaller quantities of other collagens with different functions. Aggrecan and versican are large, aggregating proteoglycans, whose main function is to provide compression resistance to the tendon by increasing water content [2]. Decorin, biglycan, fibromodulin, lumican, and osteoglycin are small leucine-rich proteoglycans, which play a role in fibril assembly and tendon integrity [3,4,5], while syndecans are transmembrane heparan sulfate proteoglycans, whose roles in tendons have remained elusive [6]. Thus, while collagen 1 fibers are responsible for the tensile strength of tendons, proteoglycans are regulating their viscoelastic properties. Tenoblasts and tenocytes constitute 90–95% of tendon cells and are fibroblast-like cells responsible for the production of collagen and other ECM components [1]. Tenocytes respond to mechanical load of tendons by altering the expression of ECM proteins, thereby modulating tendon structure, composition, and mechanical properties [7].

Cerebral palsy (CP) is a group of disorders of the development of movement and posture, causing activity limitations, which are attributed to non-progressive brain lesions that occurred in the developing fetus or infant [8]. CP is the most common chronic movement disorder in children with a prevalence of 1.5 to 4 per 1000 live births [9,10,11,12,13,14,15], and one of the most disabling and costly chronic disorders of children and adults. Although CP is the result of a non-progressive lesion of the developing brain, it leads to progressive alterations of the musculoskeletal system, which can manifest with different clinical presentations, such as spasticity, dyskinesia, dystonia, and hypertonia, often leading to the development of fixed contractures, i.e., a permanent shortening and stiffening of the muscle-tendon unit, resulting in the loss of joint motility (reviewed in [16,17]). Patients are diagnosed in respect to the anatomical distribution of their deformity as monoplegic, hemiplegic, diplegic, or tetraplegic (reviewed in [16]) as well as the degree of impairment of motor function as classified by the 5-level Gross Motor Function Classification System (GMFCS), based on observation of self-initiated movements, with emphasis on sitting, walking, and wheeled mobility [18]. Involvement of only one limb is referred to as monoplegia, unilateral involvement of an upper and lower limb is referred to as hemiplegia, predominant lower limb involvement is referred to as diplegia, involvement of the lower limbs and one upper limb is referred to as triplegia, and involvement of all four limbs and the trunk is referred to as quadriplegia. Various treatment strategies, including physiotherapy, pharmacological interventions, neurectomy, and orthosis, are used to reduce spasticity, limit pain, and prevent contractures [16,17]. Nevertheless, due to muscle hyperactivity (hypertonia), most CP patients eventually develop fixed contractures, necessitating tendon lengthening surgery to restore the joint range of motion and relieve symptoms. In previous studies, our coworkers found that tendons of CP patients show ECM remodeling [19] and altered expression of selected genes related to collagen turnover [20,21]. However, the effect of CP on genome-wide gene expression in tendons has not been studied and it remains poorly understood how the exposure of tendons to the spasticity-induced increased mechanical loading and functional demands in CP patients affect tendon homeostasis at the molecular level. In the present study, we performed total RNA-sequencing (RNA-Seq) analysis on semitendinosus tendons from diplegic (GMFCS 2–4) and tetraplegic (GMFCS 5) CP patients undergoing tendon lengthening surgery compared to age-matched controls undergoing cruciate ligament (ACL) reconstruction.

## 2. Materials and Methods

### 2.1. Sample Collection and Preparation

A cohort of 30 children were included in the study: 12 children with diplegic CP (GMFCS 2–3; 10 males and 2 females; mean age: 13.9 years), 13 children with tetraplegic CP (GMFCS 5; 8 males and 5 females; mean age: 12.6 years), and 5 typically developing pediatric patients as control patients (4 males and 1 female; mean age: 17.9). All CP patients included in the study had perinatal hypertonic CP, characterized by muscle stiffness and spasticity. None of the patients had hereditary CP. The characteristics of the subjects are listed in Table 1. Semitendinosus tendon biopsies were obtained during surgery of CP patients undergoing tendon lengthening or control patients undergoing ACL reconstruction. Biopsies were immediately frozen on dry ice and stored at –80 °C for further processing.

### 2.2. RNA Extraction

For extraction of tendon RNA, tendons were first powderized with a hammer on dry ice after which TRIzol Reagent (Thermo Fisher Scientific, Segrate (Milan), Italy) was added and tendons were homogenized using a TissueLyser II (Qiagen). RNA was subsequently extracted using the RNA-ZOL Direct Clean-up Kit (Fisher Molecular Biology, Rome, Italy) following the instructions of the manufacturer. After DNase I treatment, RNA purity and concentration were evaluated using a NanoDrop 1000 spectrophotometer (Thermo Fisher Scientific) and a Qubit Fluorometer (Thermo Fisher Scientific). RNA quality was analyzed on an Agilent Tape Station 4200 system and the RNA integration number (RIN) varied from 2.7–9.1.

### 2.3. RNA-Seq Analysis

Indexed sequencing libraries were generated from 140 ng of tendon RNA using the KAPA RNA HyperPrep with RiboErase (Roche), which allows for library construction from partially degraded samples. Paired-end multiplexed sequencing of libraries to generate reads of 150 bp (PE 2 × 250) was performed on a NovaSeq 6000 instrument (Illumina Inc., San Diego, CA, USA). For each sample, 22–40 million reads were obtained. RNA-Seq samples were first demultiplexed and FASTQ files were created from BCL files using bcl2fastq v2.17.1.14 software (Illumina Inc.). Quality control and assessment were performed on FASTQ files using FastQC v0.11.9 (http://www.bioinformatics.babraham.ac.uk/projects/fastqc). Paired-end reads of 150 bp were aligned to the Gencode Human reference genome (build GRCh38) using STAR v2.7.2b [22]. Annotation of genes was performed using the biomaRt package v2.54 [23] based on annotations from Ensembl release 110. The raw read counts were normalized with TMM implemented in the edgeR v3.40 package [24] in R/Bioconductor [25] and less-expressed genes were filtered out with the filterByExpr (min.count = 6) function. Differential expression analysis of read counts was performed using the voom, lmFit, and eBayes (robust = T) functions of the limma v3.46 package [26] in R. Significantly differentially expressed genes were chosen based on a false discovery rate (FDR) < 0.1 and |log2 fold change (FC)| ≥ 0.4. The sva v3.38 package [27] was used to estimate artifacts (n.sv = 1) and correct the CPM values. Hierarchical clustering of significantly modulated genes was performed using hclust and dist functions on sva corrected log2CPM in R. Clustering was performed with the ward.D2 method and Euclidean distance to generate a heatmap using pheatmap v1.0.12 (https://cran.r-project.org/web/packages/pheatmap/index.html) and scaling the rows. Principal components analysis (PCA) was performed using the PCA function of the FactoMineR v2.7 [28] package in R. The Venn diagrams were obtained using the R package VennDiagram v1.7.3 (https://cran.r-project.org/web/packages/VennDiagram/index.html).

Raw data files for the RNA-Seq analysis have been deposited in the NCBI BioProject database (https://www.ncbi.nlm.nih.gov/bioproject/) on 3 August 2023 under accession code PRJNA1004310.

### 2.4. Gene Ontology and Pathway Analysis

For gene enrichment analysis, the gene ontology (GO) Biological Process 2021 and Reactome 2022 databases within EnrichR [29] were used to find significant enriched GO terms and pathways with an adjusted *p* value ≤ 0.05.

### 2.5. Quantitative Real-Time PCR (qRT-PCR)

First-strand cDNA synthesis was performed using the High Capacity cDNA Reverse Transcription kit (Thermo Fisher Scientific), whereafter qRT-PCR was performed in triplicate with custom-designed oligos (see Table S1) using the SYBR Select Master Mix (Thermo Fisher Scientific). Relative expression analysis was performed using the ∆∆Ct method with *GAPDH* as reference gene.

### 2.6. Statistical Analysis

For qRT-PCR, the statistical comparisons between the tree groups were performed using one-way ANOVA with Tukey’s multiple comparisons test. The Shapiro–Wilk test was performed to confirm normal distribution in each group; Anderson–Darlin, D’Agostino, Shapiro–Wilk, and Kolmogorov–Smirnov tests to verify normality of residuals, and Brown–Forsythe and Barlett’s tests to check for clustering and heteroscedasticity of residuals. When necessary, data were log-transformed to meet ANOVA assumptions. Statistical analysis was performed using Prism v9.1.1 (GraphPad) software.

## 3. Results

**Figure 1:**
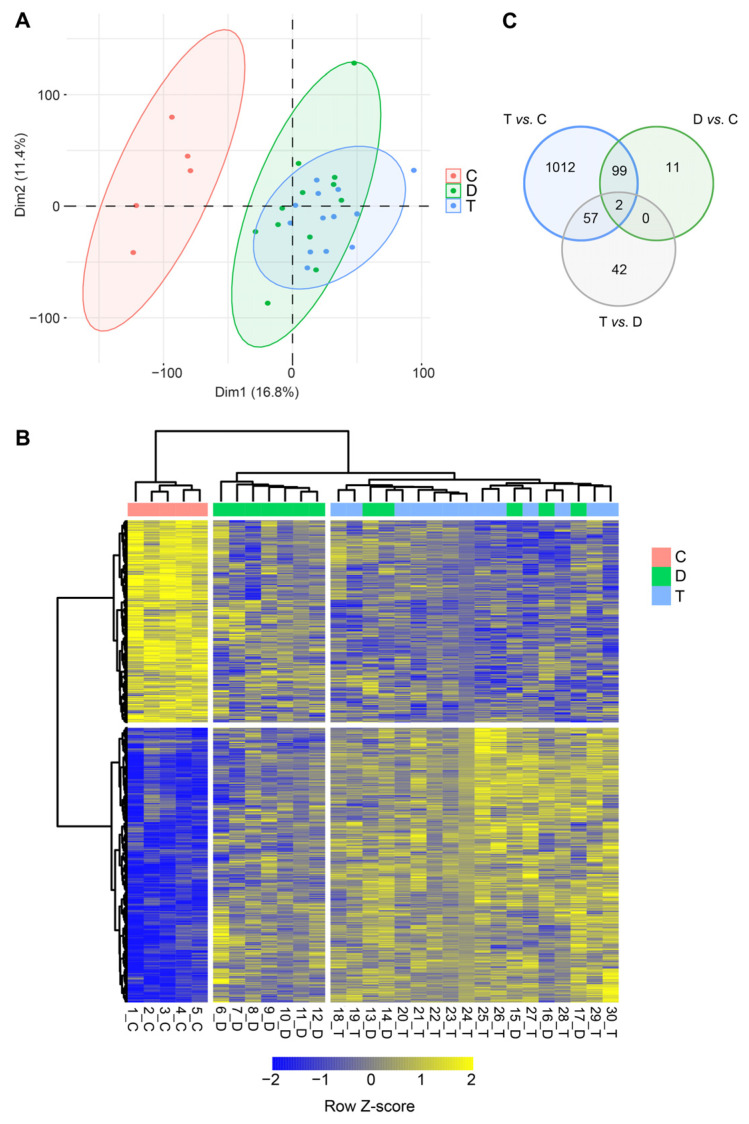
RNA-Seq analysis on semitendinosus tendons from 12 diplegic CP (D), 12 tetraplegic CP (T), and 5 control (C) patients. (**A**) Principal components analysis (PCA) based on transcriptional profiles with ellipses of confidence intervals set at 0.9 for each group. (**B**) Heat map of unsupervised hierarchical clustering of 1223 differentially expressed protein-coding genes (FDR ≤ 0.1; |log2FC| ≥ 0.4). (**C**) Venn diagram of the pairwise differential expression (DE) analysis (FDR ≤ 0.1; |log2FC| ≥ 0.4).

**Figure 2:**
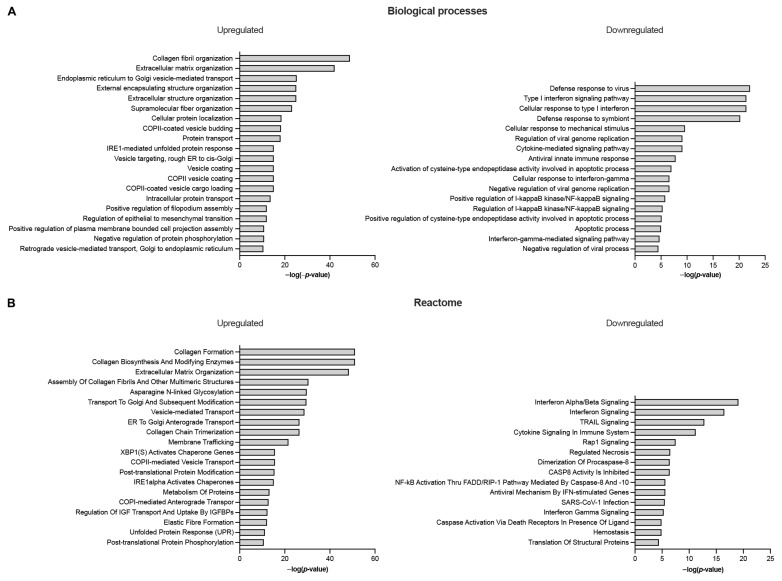
EnrichR analysis for upregulated and downregulated genes in semitendinosus tendons from tetraplegic CP (T) patients vs. control (C) patients (FDR ≤ 0.1; |log2FC| ≥ 0.4). (**A**) Gene ontology (GO) analysis for biological processes, and (**B**) Reactome analysis (adjusted *p* value ≤ 0.05). For upregulated genes, the 20 most significantly enriched GO terms and pathways are shown.

**Figure 3:**
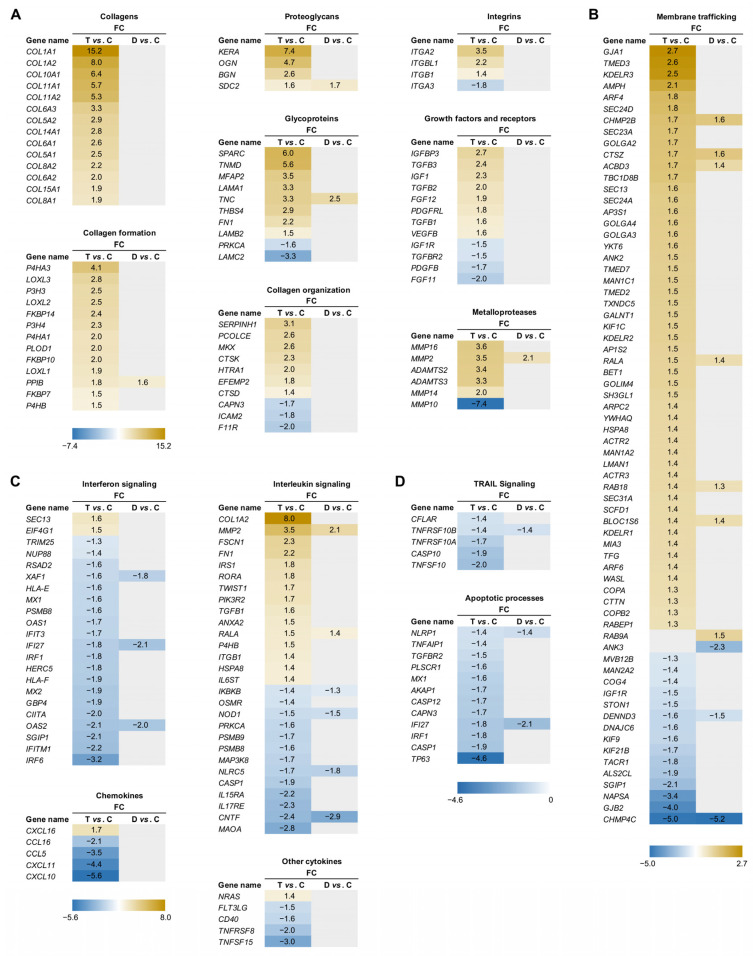
Differentially expressed genes in semitendinosus tendons from tetraplegic (T) and diplegic (D) CP patients vs. control (C) patients involved in ECM organization (**A**), membrane trafficking (**B**), cytokine signaling (**C**), and apoptotic processes (**D**) (FDR ≤ 0.1; |log2FC| ≥ 0.4).

**Figure 4:**
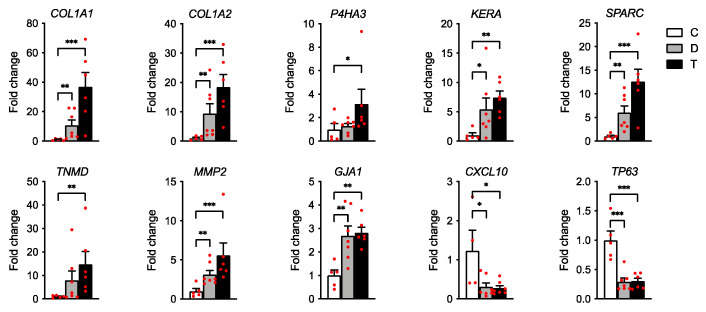
qRT-PCR analysis for selected differentially expressed genes on tendon RNA from tetraplegic (T) and diplegic (D) CP patients vs. control (C) patients. Data are represented as mean ± standard error of the mean (SEM) (*n* = 5–7 biological replicates and 3 technical replicates per group). Data were normalized to *GAPDH*. * *p* < 0.05; ** *p* < 0.01; *** *p* < 0.001; one-way analysis of variance (ANOVA) with Tukey’s multiple comparisons test.

Total RNA-Seq analysis was performed on semitendinosus tendons from 12 diplegic CP, 13 tetraplegic CP, and 5 control patients. Unfortunately, due to muscle contamination five control samples had to be excluded. Principal component analysis (PCA) (Figure 1A) and hierarchical clustering showed clustering of the control group distant from the CP groups (Figure 1A,B). A total of 1170 genes (676 upregulated and 494 downregulated) were differentially expressed in tetraplegic vs. control patients, 112 in diplegic CP vs. control patients (61 upregulated and 51 downregulated), and 101 (70 upregulated and 31 downregulated) in tetraplegic vs. diplegic CP patients. As illustrated in the Venn diagram in Figure 1C, 101 genes (58 upregulated and 43 downregulated) were commonly altered in diplegic and tetraplegic CP patients, while 1068 and 11 genes were differentially expressed specifically in tetraplegic vs. control patients and diplegic CP vs. control patients, respectively. Thus, most genes that were differentially expressed in diplegic CP patients were altered also in tetraplegic CP patients. The full lists of differentially expressed (DE) genes are shown in Supplementary Materials Tables S2–S4. For genes that were differentially expressed in tetraplegic CP vs. control patients, gene ontology analysis for biological processes as well as Reactome pathway analysis were performed (Figure 2 and Tables S5–S8). For upregulated genes, there was an enrichment for genes involved in collagen formation, ECM organization, and categories related to vesicle-mediated transport (Figure 2A,B, left), while for downregulated genes, genes related to cytokine and apoptotic signaling, including TNF-related apoptosis-inducing agent (TRAIL) signaling, were most significantly affected (Figure 2A,B, right). The differentially expressed genes in diplegic CP patients did not show significant enrichment for genes involved to any specific process. Differentially expressed genes in tetraplegic CP patients vs. control patients involved in ECM organization, vesicle-mediated transport, cytokine signaling, and apoptotic signaling are listed in Figure 3. Many genes encoding collagens or involved in collagen formation and organization were upregulated, including proteoglycans, glycoproteins, integrins, and growth factors (Figure 3A). Increased expression of several genes encoding metallopeptidases (MMPs), involved in ECM turnover, was also found, including *MMP2*, which is the most expressed MMP in tendons. Furthermore, many genes involved in membrane trafficking were upregulated (Figure 3B). Among the downregulated genes were many genes involved in cytokine signaling, including interferon signaling, interleukin signaling, and chemokines (Figure 3C) as well as a number of genes related to TRAIL signaling and apoptosis (Figure 3D). The majority of these genes were not altered in the more mildly affected diplegic CP patients. The results were confirmed by quantitative qRT-PCR analysis for selected modulated genes (Figure 4).

## 4. Discussion

The present study is the first to report genome-wide expression profiling on tendons from CP patients. Consistent with our previous findings [20], increased expression of genes encoding collagens and ECM proteins was found in the tendons of tetraplegic CP (GMFCS 5) patients, but not in the less-affected diplegic CP (GMFCS 2–4) patients. The most upregulated genes were the collagen 1-encoding genes *COL1A1* and *COL1A2*, which were 15- and 8-fold upregulated, respectively. Furthermore, genes encoding both fibrillar (*COL5A1/2*, *COL11A1/2*) and non-fibrillar collagens (*COL6A1*/*2*/*3* (beaded filament-forming collagen), *COL8A1*/*2* (hexagonal network-forming collagen), *COL10A1* (hexagonal network-forming collagen), *COL14A1* (fibril-associated collagen with interrupted triple helix (FACIT)), *COL15A1* (FACIT)) were highly upregulated as shown in Figure 3A [30].

Collagen is composed of a triple helix, consisting of three α chains with the repeating (Gly-X-Y)n amino acid sequence, where X and Y are frequently proline or hydroxyproline but can be any amino acid [30]. While some collagens contain three identical α chains, others contain two or three different chains referred to as α1, α2, and α3. In tendons, fibrillar collagens are bundled into fibrils, which form larger fibers. During collagen biosynthesis, collagen undergoes post-translational modification by hydroxylation of proline residues into hydroxyproline by prolyl 3-hydroxylases (P3H1, P3H2, P3H3, P3H4) and prolyl 4-hydroxylases (a tetramer composed of two α (P4H1, P4H2, P4H3) and one β (P4HB) subunit) as well as hydroxylation of lysine into hydroxylysine by lysyl hydroxylases (LH1, LH2, LH3), encoded by procollagen-lysine,2-oxoglutarate 5-dioxygenases (*PLOD1*, *PLOD2*, *PLOD3*) [31,32]. Hydroxylysine residues are often subject to glycosylation and P4HB is responsible for the formation of disulfide bands between the α chains. Peptidyl-prolyl *cis-trans* isomerases (FKBP10, FKBP14, PPIB) catalyze the *cis-trans* isomerization of peptide bonds, which is a rate limiting step for triple helix formation. After secretion, lysyl oxidases (LOX, LOXLs) oxidize hydroxylysine residues to induce cross-link formation [33], which provide the collagen fibrils with mechanical stability and tensile strength as well as contribute to the stiffness of the collagen fibril. In tendons of tetraplegic CP patients, genes involved in all steps of collagen formation and cross-linking were upregulated, including *P3H3, P3H4*, *P4HA1*, *P4HA3*, *P4HB*, *PLOD1*, *FKBP10*, *FKBP14*, *PPIB*, *LOXL1*, *LOXL2*, and *LOXL3*, while only *PPIB* was increased in diplegic CP patients. This suggests that collagen synthesis is strongly induced in tetraplegic CP patients.

In tetraplegic CP patients, genes encoding the small leucine-rich proteoglycans biglycan (*BGN*), keratocan (*KERA*), and osteoglycin (*OGN*) were highly upregulated by 2.4-, 7.4-, and 4.7-fold, respectively. Biglycan was recently shown to be involved in the maintenance of tendon structure and mechanics in mature tendons [34] and osteoglycin has been implicated in collagen fibrillar organization and tendon mechanical function [5,35], while the role of keratocan has remained elusive [36]. Decorin, which constitutes about 80% of the total amount of small leucine-rich proteoglycans in tendons, was not affected. This may be explained by a recent study, which demonstrated that while decorin plays a more important role than biglycan (the second most abundant small leucine-rich proteoglycan in tendons) in the modulation of collagen fibril structure and viscoelastic mechanics during tendon development, biglycan is more important for the maintenance of tendon structure and mechanical properties during homeostasis in mature tendons [34]. While the expression of none of the genes encoding small leucine-rich proteoglycans was altered in diplegic CP patients, *SDC2,* encoding the transmembrane heparan sulfate proteoglycan syndecan-2, was upregulated in both diplegic and tetraplegic CP patients by 1.7- and 1.6-fold, respectively.

Several genes encoding glycoproteins, including tenascin-C (*TNC*), fibronectin 1 (*FN1*), tenomodulin (*TNMD*), secreted protein acidic and rich in cysteine (*SPARC*), and thrombospondin 4 (*THBS4*) were upregulated in tetraplegic CP patients. In particular, *SPARC* and *THBS4* were strongly upregulated by 6.0- and 5.6-fold, respectively. SPARC has been shown to be essential for load-induced tendon tissue maturation and homeostasis, affecting ECM composition and tendon biomechanical properties [37], while tenomodulin plays a role in tenocyte proliferation and collagen fibril maturation [38]. Thrombospondin 4, encoded by *THBS4*, which was 2.9-fold upregulated, is involved in the organization of collagen fibrils [38], while tenascin-C and fibronectin, whose genes were 3.3- and 2.2-fold upregulated, respectively, both contribute to the mechanical stability of the ECM [39,40]. Among the glycoproteins, only *TNC* was upregulated (2.2-fold) in diplegic CP patients. In tetraplegic CP patients, alterations in the expression of genes encoding different subunits of the large multidomain heterotrimeric glycoprotein laminin was also found. Laminin is located in the basement membrane, where it interacts with collagen type IV, integrins, and dystroglycans, and play important roles in cell adhesion, differentiation, and migration [41]. *LAMA1* (encoding laminin α1) and *LAMB2* (encoding laminin β2) were 3.3- and 1.5-fold upregulated, respectively, while *LAMC2* (encoding laminin γ2) was 3.3-fold downregulated.

Altered mRNA expression of several integrins was also found in the tendons of tetraplegic CP patients, including *ITGA2*, *ITGB1*, and *ITGBL1*, which were 3.5-, 1.4-, and 2.2-fold upregulated, respectively, as well as *ITGA3,* which was 1.8-fold downregulated. Integrins are heterodimeric transmembrane receptors, which play a major role in linking the ECM to the cytoskeleton [42]. It is believed that integrins can sense and transmit mechanical stimuli from the ECM to tenocytes, thereby triggering intracellular signaling pathways leading to adaptive regulation of gene expression. In particular, mechanical stretch was found to activate the AKT/mTOR pathway via β1 integrin, thereby regulating collagen expression [43]. Also, *GJA1*, encoding connexin 43, a gap junction protein with an important role in the communications between tenocytes allowing for the passage of passage of free metabolites and ions [44], was 2.7-fold upregulated in tetraplegic CP patients.

The expression of a number of genes encoding growth factors were increased in tendons of tetraplegic CP patients, including integrin-like growth factor 1 (*IGF1*), integrin-like growth factor binding protein 3 (*IGFBP3*), fibroblast growth factor 12 (*FGF12*), transforming growth factors (*TGFB1*, *TGFB2*, *TGFB3*), and vascular endothelial growth factor B (*VEGFB*), while *FGF11* was downregulated (see Figure 3A). IGF1 has been shown to be required for adult tendon growth in response to increased mechanical overload through stimulation of tenocyte proliferation and protein synthesis [45]. Similarly, TGFβ was shown to promote collagen synthesis and matrix remodeling during tendon healing, thereby enhancing mechanical strength [46]. On the other hand, excessive TGFβ activation as a result of mechanical overload or repetitive mechanical loading can lead to tendinopathy [47]. VEGF promotes angiogenesis and increases vascular permeability and is important for neovascularization during tendon healing [48]. Also, the *MKX* gene encoding Mohawk, an atypical homeobox transcription factor involved in postnatal tendon maturation [49,50] and critical for the tendon response to mechanical stimuli [51], was 2.6-fold upregulated in tetraplegic CP patients.

Among the differentially expressed genes in the tendons of tetraplegic CP patients were many genes encoding metalloproteases, including matrix metalloproteinases (*MMP2*, *MMP10*, *MMP14*, and *MMP16*) and members of the ADAMTS (a disintegrin and metalloproteinase with thrombospondin motifs) protein family (*ADAMTS2*, *ADAMTS3*, *ADAMTS17*, and *ADAMTSL2*). Metalloproteases are important for the turnover of the ECM, which is a finely balanced, dynamic process of protein synthesis and degradation taking place at a low rate during homeostasis and at higher rates during conditions of inflammation, tissue damage, or increased mechanical load [52]. Metalloproteinases mediate the proteolytic degradation of components of the ECM and are regulated by tissue inhibitors of metalloproteinases (TIMS). While ECM turnover allows for tendon adaptation in response to altered mechanical loading conditions, an imbalance in the activities of MMPs and TIMS can lead to pathological conditions [53]. MMP2, MMP14, and MMP16, whose mRNA levels were 3.5-, 2.0-, and 3.6-fold upregulated, respectively, are able to cleave fibrillar collagen I, while MMP10, whose gene was 7.4-fold downregulated, does not cleave collagen I [53], but degrades various components of the ECM, including proteoglycans, fibronectin, and collagen III-V [54] as well as activates other MMPs [55]. ADAMTS2 and ADAMTS3, which were 3.4- and 3.3-fold upregulated at the mRNA level, respectively, are involved in fibrillar collagen maturation though cleavage of the N-terminal propeptide of procollagens [56,57]. Also, *PCOLCE*, encoding procollagen C-endopeptidase enhancer, which enhances the activity of procollagen C proteinases cleaving the C-terminal propeptide of procollagens, was 2.6-fold upregulated [58]. In addition, mRNA encoding ADAMTS17, whose function in tendons has remained elusive, was 2.5-fold upregulated. On the other hand, transcript levels of ADAMTSL2, which belongs to the subfamily of ADAMTS-like proteins without catalytic activity and is involved in the modulation of microfibril formation, were 2.1-fold reduced [59]. Among the metalloproteases, only MMP2 was upregulated (2.1-fold) in diplegic CP patients.

Consistent with increased protein turnover and remodeling in tetraplegic CP patients, Reactome analysis and gene ontology analysis for biological processes revealed an enrichment for upregulated genes involved in vesicle-mediated transport (Figure 2A, B, left). Among the downregulated genes, there was an enrichment for genes involved in interferon and cytokine signaling, including a number of chemokines (Figure 2A, B, right, Figure 3C). In particular, *CCL5*, *CCL16*, *CXCL10*, and *CXCL11* were strongly downregulated by, respectively, 3.5-, 2.1-, 5.6-, and 4.4-fold, while *CXCL16* was 1.7-fold upregulated. Thus, the higher mechanical load experienced by tendons of CP patients does not appear to be associated with inflammation. Furthermore, apoptosis does not seem to be activated as there was an enrichment for downregulated genes associated with TRAIL signaling and apoptotic processes (Figure 3D).

The results of our RNA-Seq analysis are consistent with increased collagen synthesis and turnover in tendons from tetraplegic CP patients as a consequence of the spasticity-induced chronic mechanical stimulation. Similarly, increased expression of genes involved in ECM organization has been reported in skeletal muscle of CP patients [60,61,62]. Many of the changes in gene expression observed in CP patients are similar to those observed in the tendons of patients with tendinopathy, but there are important differences suggesting that the altered gene expression in tetraplegic CP patients may be adaptive rather than pathological as in tendinopathy. As in tendinopathy patients, the expression of many collagens was increased. However, while the mRNA expression of a number of collagens was 1.6–2.7-fold upregulated in tendinopathy patients, *COL1A1*, *COL1A2, COL10A1*, and *COL11A1/2* were 5.3–15.2-fold upregulated in tetraplegic CP patients, suggesting strongly increased collagen synthesis. In regard to the expression of metalloprotease-encoding genes, increased expression of *MMP2*, *MMP14*, *MMP16*, *ADAMTS2*, *ADAMTS3* as well as downregulation of *MMP10* has also been found in tendinopathy [63,64]. Also, upregulation of genes encoding proteoglycans (*BGN*) glycoproteins (*TNC*, *FN1*, *SPARC*), integrins (*ITGB1*), and growth factors has been reported in tendinopathy patients [64,65,66]. In addition, although the role of inflammation in tendinopathy remains controversial [67,68,69,70], a number of genes encoding pro-inflammatory cytokines have been reported to be upregulated in tendinopathy [64,66], while many chemokines and interferons were downregulated in tetraplegic CP patients (see Figure 3C), perhaps as a reflection of regulatory immune mechanisms involving tenocytes and immune cells. Moreover, whereas increased expression of markers of apoptosis was found in tendinopathy [66], many apoptosis-related genes were downregulated in tetraplegic CP patients. In addition, an enrichment for upregulated genes associated with vesicle-mediated transport has not been reported in tendinopathy patients. Overall, our results indicate that tetraplegic CP patients show increased ECM turnover with an increased net synthesis of collagen to allow for reorganization of the ECM as an adaptive response to increased mechanical loads and functional demands as a result of spasticity. This is similar to observations after exercise training, where ECM remodeling has been found to result in reduced tendon stress and increased tendon stiffness and tensile strength [71,72,73,74,75,76,77], likely generating more resistant tendons to support higher loads. The absence of pro-inflammatory cytokines and markers of apoptosis in CP patients compared to tendinopathy patients could be due to the difference in stress stimuli, being submaximal and continuous in the former and over maximal and discontinuous in the latter. Although tendon remodeling in CP patients does not appear to be directly pathological, increased tendon stiffness and shortening in CP patients as a result of continuous mechanical stimulation is an important issue in clinical practice, where tendon extension surgery is often required to restore joint mobility. In clinal practice, various kinds of muscle relaxants and neuromuscular blocking agents are also often used to reduce spasticity and consequently decrease pathological stimulation of tendons and prevent the development of contractures [16,17]. In future studies, it would be interesting to perform mechanical tension studies and histological analyses of patients with different severity of CP to determine whether they show increased tendon stiffness and cross-sectional area as observed in tendons from long-distance runners [78,79] and after high resistance training [72,73,75,76,80]. The many fewer gene expression changes in diplegic compared to tetraplegic CP patients implies that tendons of patients with a less severe form of CP are not affected by the disease to an extent that induces major tendon remodeling, consistent with the fact that only intense exercise is associated with substantial tendon remodeling [72,78,80]. This is also consistent with our personal observations in the clinic, where increased tendon stiffness is generally more evident in tetraplegic CP patients compared to diplegic CP patients.

In conclusion, the present study provides new insights into the alterations in gene expression in CP patients as a consequence of the chronically increased mechanical loads due to muscle hyperactivity. A better understanding of the molecular alterations in the tendons of CP patients during the course of the disease may be beneficial for disease monitoring and lead to the identification of novel therapeutic targets and development of better treatment strategies to alleviate symptoms and improve quality of life in CP patients.

## Figures and Tables

**Table 1:** Patient characteristics.

Patient	Gender	Age	Type
1	F	15.8	C
2	M	26.4	C
3	M	14.6	C
4	M	16.1	C
5	M	16.8	C
6	M	21.1	D
7	M	15.5	D
8	M	17.1	D
9	M	15.0	D
10	F	5.6	D
11	M	16.5	D
12	M	10.5	D
13	M	11.0	D
14	M	15.7	D
15	F	11.4	D
16	M	17.1	D
17	M	10.1	D
18	M	11.5	T
19	F	11.0	T
20	F	14.5	T
21	M	16.2	T
22	M	4.9	T
23	F	14.0	T
24	M	14.9	T
25	F	12.8	T
26	F	11.6	T
27	M	13.3	T
28	M	9.2	T
29	M	16.2	T
30	M	13.0	T

M, male; F, female; C, control, D, diplegic; T, tetraplegic.

## Data Availability

The data presented in this study are openly available in the NCBI BioProject database (https://www.ncbi.nlm.nih.gov/bioproject/) deposited on 3 August 2023 under accession code PRJNA1004310. The data presented in this study are available in Tables S1–S7.

## Supplementary Materials

The following supporting information can be downloaded at: https://www.mdpi.com/article/10.3390/biomedicines11112918/s1. Table S1: Oligos used for quantitative real-time PCR (qRT-PCR). Table S2: Differentially expressed genes in tetraplegic CP patients vs. control patients. Table S3: Differentially expressed genes in diplegic CP patients vs. control patients. Table S4: Differentially expressed genes in tetraplegic CP patients vs. diplegic CP patients. Table S5: Gene ontology analysis for biological processes for upregulated genes in tetraplegic CP patients vs. control patients. Table S6: Gene ontology analysis for biological processes for downregulated genes in tetraplegic CP patients vs. control patients. Table S7: Reactome analysis for upregulated genes in tetraplegic CP patients vs. control patients. Table S8: Reactome analysis for downregulated genes in tetraplegic CP patients vs. control patients.

## References

[1] Kannus P. (2000). Structure of the tendon connective tissue. Scand. J. Med. Sci. Sports.

[2] Vogel K.G. (2004). What happens when tendons bend and twist? Proteoglycans. J. Musculoskelet. Neuronal Interact..

[3] Ezura Y., Chakravarti S., Oldberg A., Chervoneva I., Birk D.E. (2000). Differential expression of lumican and fibromodulin regulate collagen fibrillogenesis in developing mouse tendons. J. Cell Biol..

[4] Dunkman A.A., Buckley M.R., Mienaltowski M.J., Adams S.M., Thomas S.J., Satchell L., Kumar A., Pathmanathan L., Beason D.P., Iozzo R.V. (2013). Decorin expression is important for age-related changes in tendon structure and mechanical properties. Matrix Biol..

[5] Boote C., Ma Q., Goh K.L. (2023). Age-dependent mechanical properties of tail tendons in wild-type and mimecan gene-knockout mice—A preliminary study. J. Mech. Behav. Biomed. Mater..

[6] Gopal S., Arokiasamy S., Pataki C., Whiteford J.R., Couchman J.R. (2021). Syndecan receptors: Pericellular regulators in development and inflammatory disease. Open Biol..

[7] Wang J.H. (2006). Mechanobiology of tendon. J. Biomech..

[8] Bax M., Goldstein M., Rosenbaum P., Leviton A., Paneth N., Dan B., Jacobsson B., Damiano D. (2005). Proposed definition and classification of cerebral palsy, April 2005. Dev. Med. Child Neurol..

[9] Paneth N., Hong T., Korzeniewski S. (2006). The Descriptive Epidemiology of Cerebral Palsy. Clin. Perinatol..

[10] Yeargin-Allsopp M., Van Naarden Braun K., Doernberg N.S., Benedict R.E., Kirby R.S., Durkin M.S. (2008). Prevalence of cerebral palsy in 8-year-old children in three areas of the United States in 2002: A multisite collaboration. Pediatrics.

[11] Surveillance of Cerebral Palsy in Europe (2002). Prevalence and characteristics of children with cerebral palsy in Europe. Dev. Med. Child Neurol..

[12] Van Naarden Braun K., Doernberg N., Schieve L., Christensen D., Goodman A., Yeargin-Allsopp M. (2016). Birth Prevalence of Cerebral Palsy: A Population-Based Study. Pediatrics.

[13] El-Tallawy H.N., Farghaly W.M., Shehata G.A., Rageh T.A., Metwally N.A., Badry R., Sayed M.A., Abd El Hamed M., Abd-Elwarth A., Kandil M.R. (2014). Cerebral palsy in Al-Quseir City, Egypt: Prevalence, subtypes, and risk factors. Neuropsychiatr. Dis. Treat..

[14] Chang M.J., Ma H.I., Lu T.H. (2015). Estimating the prevalence of cerebral palsy in Taiwan: A comparison of different case definitions. Res. Dev. Disabil..

[15] Christensen D., Van Naarden Braun K., Doernberg N.S., Maenner M.J., Arneson C.L., Durkin M.S., Benedict R.E., Kirby R.S., Wingate M.S., Fitzgerald R. (2014). Prevalence of cerebral palsy, co-occurring autism spectrum disorders, and motor functioning—Autism and Developmental Disabilities Monitoring Network, USA, 2008. Dev. Med. Child Neurol..

[16] Graham H.K., Rosenbaum P., Paneth N., Dan B., Lin J.P., Damiano D.L., Becher J.G., Gaebler-Spira D., Colver A., Reddihough D.S. (2016). Cerebral palsy. Nat. Rev. Dis. Primers.

[17] Handsfield G.G., Williams S., Khuu S., Lichtwark G., Stott N.S. (2022). Muscle architecture, growth, and biological Remodelling in cerebral palsy: A narrative review. BMC Musculoskelet. Disord..

[18] Palisano R., Rosenbaum P., Walter S., Russell D., Wood E., Galuppi B. (1997). Development and reliability of a system to classify gross motor function in children with cerebral palsy. Dev. Med. Child Neurol..

[19] Gagliano N., Menon A., Martinelli C., Pettinari L., Panou A., Milzani A., Dalle-Donne I., Portinaro N.M. (2013). Tendon structure and extracellular matrix components are affected by spasticity in cerebral palsy patients. Muscles Ligaments Tendons J..

[20] Gagliano N., Pelillo F., Chiriva-Internati M., Picciolini O., Costa F., Schutt R.C., Gioia M., Portinaro N. (2009). Expression profiling of genes involved in collagen turnover in tendons from cerebral palsy patients. Connect. Tissue Res..

[21] Gagliano N., Pelillo F., Grizzi F., Picciolini O., Gioia M., Portinaro N. (2007). Gene expression profile of extracellular matrix of tendons in cerebral palsy. Dev. Med. Child Neurol..

[22] Dobin A., Davis C.A., Schlesinger F., Drenkow J., Zaleski C., Jha S., Batut P., Chaisson M., Gingeras T.R. (2013). STAR: Ultrafast universal RNA-seq aligner. Bioinformatics.

[23] Durinck S., Spellman P.T., Birney E., Huber W. (2009). Mapping identifiers for the integration of genomic datasets with the R/Bioconductor package biomaRt. Nat. Protoc..

[24] Robinson M.D., McCarthy D.J., Smyth G.K. (2010). edgeR: A Bioconductor package for differential expression analysis of digital gene expression data. Bioinformatics.

[25] Gentleman R.C., Carey V.J., Bates D.M., Bolstad B., Dettling M., Dudoit S., Ellis B., Gautier L., Ge Y., Gentry J. (2004). Bioconductor: Open software development for computational biology and bioinformatics. Genome Biol..

[26] Ritchie M.E., Phipson B., Wu D., Hu Y., Law C.W., Shi W., Smyth G.K. (2015). *limma* powers differential expression analyses for RNA-sequencing and microarray studies. Nucleic Acids Res..

[27] Leek J.T., Johnson W.E., Parker H.S., Jaffe A.E., Storey J.D. (2012). The sva package for removing batch effects and other unwanted variation in high-throughput experiments. Bioinformatics.

[28] Le S., Josse J., Husson F. (2008). FactoMineR: An R package for multivariate analysis. J. Stat. Softw..

[29] Kuleshov M.V., Jones M.R., Rouillard A.D., Fernandez N.F., Duan Q., Wang Z., Koplev S., Jenkins S.L., Jagodnik K.M., Lachmann A. (2016). Enrichr: A comprehensive gene set enrichment analysis web server 2016 update. Nucleic Acids Res..

[30] Ricard-Blum S. (2011). The collagen family. Cold Spring Harb. Perspect. Biol..

[31] Heino J. (2007). The collagen family members as cell adhesion proteins. Bioessays.

[32] Gjaltema R.A., Bank R.A. (2017). Molecular insights into prolyl and lysyl hydroxylation of fibrillar collagens in health and disease. Crit. Rev. Biochem. Mol. Biol..

[33] Ellingson A.J., Pancheri N.M., Schiele N.R. (2022). Regulators of collagen crosslinking in developing and adult tendons. Eur. Cells Mater..

[34] Beach Z.M., Bonilla K.A., Dekhne M.S., Sun M., Adams T.H., Adams S.M., Weiss S.N., Rodriguez A.B., Shetye S.S., Birk D.E. (2022). Biglycan has a major role in maintenance of mature tendon mechanics. J. Orthop. Res..

[35] Tasheva E.S., Koester A., Paulsen A.Q., Garrett A.S., Boyle D.L., Davidson H.J., Song M., Fox N., Conrad G.W. (2002). Mimecan/osteoglycin-deficient mice have collagen fibril abnormalities. Mol. Vis..

[36] Rees S.G., Waggett A.D., Kerr B.C., Probert J., Gealy E.C., Dent C.M., Caterson B., Hughes C.E. (2009). Immunolocalisation and expression of keratocan in tendon. Osteoarthr. Cartil..

[37] Wang T., Wagner A., Gehwolf R., Yan W., Passini F.S., Thien C., Weissenbacher N., Lin Z., Lehner C., Teng H. (2021). Load-induced regulation of tendon homeostasis by SPARC, a genetic predisposition factor for tendon and ligament injuries. Sci. Transl. Med..

[38] Alexandrov V.P., Naimov S.I. (2016). A Prospectus of Tenomodulin. Folia Med..

[39] Wang J.H., Guo Q., Li B. (2012). Tendon biomechanics and mechanobiology—A minireview of basic concepts and recent advancements. J. Hand Ther..

[40] Jones F.S., Jones P.L. (2000). The tenascin family of ECM glycoproteins: Structure, function, and regulation during embryonic development and tissue remodeling. Dev. Dyn..

[41] Halper J., Kjaer M. (2014). Basic components of connective tissues and extracellular matrix: Elastin, fibrillin, fibulins, fibrinogen, fibronectin, laminin, tenascins and thrombospondins. Adv. Exp. Med. Biol..

[42] Docheva D., Popov C., Alberton P., Aszodi A. (2014). Integrin signaling in skeletal development and function. Birth Defects Res. C Embryo Today.

[43] Mousavizadeh R., Hojabrpour P., Eltit F., McDonald P.C., Dedhar S., McCormack R.G., Duronio V., Jafarnejad S.M., Scott A. (2020). β1 integrin, ILK and mTOR regulate collagen synthesis in mechanically loaded tendon cells. Sci. Rep..

[44] Maeda E., Ye S., Wang W., Bader D.L., Knight M.M., Lee D.A. (2012). Gap junction permeability between tenocytes within tendon fascicles is suppressed by tensile loading. Biomech. Model. Mechanobiol..

[45] Disser N.P., Sugg K.B., Talarek J.R., Sarver D.C., Rourke B.J., Mendias C.L. (2019). Insulin-like growth factor 1 signaling in tenocytes is required for adult tendon growth. FASEB J..

[46] Hou Y., Mao Z., Wei X., Lin L., Chen L., Wang H., Fu X., Zhang J., Yu C. (2009). The roles of TGF-beta1 gene transfer on collagen formation during Achilles tendon healing. Biochem. Biophys. Res. Commun..

[47] Wang X., Liu S., Yu T., An S., Deng R., Tan X., Crane J., Zhang W., Pan D., Wan M. (2022). Inhibition of Integrin alphavbeta6 Activation of TGF-beta Attenuates Tendinopathy. Adv. Sci..

[48] Liu X., Zhu B., Li Y., Liu X., Guo S., Wang C., Li S., Wang D. (2021). The Role of Vascular Endothelial Growth Factor in Tendon Healing. Front. Physiol..

[49] Liu W., Watson S.S., Lan Y., Keene D.R., Ovitt C.E., Liu H., Schweitzer R., Jiang R. (2010). The atypical homeodomain transcription factor Mohawk controls tendon morphogenesis. Mol. Cell. Biol..

[50] Ito Y., Toriuchi N., Yoshitaka T., Ueno-Kudoh H., Sato T., Yokoyama S., Nishida K., Akimoto T., Takahashi M., Miyaki S. (2010). The Mohawk homeobox gene is a critical regulator of tendon differentiation. Proc. Natl. Acad. Sci. USA.

[51] Kayama T., Mori M., Ito Y., Matsushima T., Nakamichi R., Suzuki H., Ichinose S., Saito M., Marumo K., Asahara H. (2016). Gtf2ird1-Dependent Mohawk Expression Regulates Mechanosensing Properties of the Tendon. Mol. Cell. Biol..

[52] Riley G. (2008). Tendinopathy—From basic science to treatment. Nat. Clin. Pr. Rheumatol..

[53] Sbardella D., Tundo G.R., Fasciglione G.F., Gioia M., Bisicchia S., Gasbarra E., Ippolito E., Tarantino U., Coletta M., Marini S. (2014). Role of metalloproteinases in tendon pathophysiology. Mini Rev. Med. Chem..

[54] Galloway W.A., Murphy G., Sandy J.D., Gavrilovic J., Cawston T.E., Reynolds J.J. (1983). Purification and characterization of a rabbit bone metalloproteinase that degrades proteoglycan and other connective-tissue components. Biochem. J..

[55] Nicholson R., Murphy G., Breathnach R. (1989). Human and rat malignant-tumor-associated mRNAs encode stromelysin-like metalloproteinases. Biochemistry.

[56] Fernandes R.J., Hirohata S., Engle J.M., Colige A., Cohn D.H., Eyre D.R., Apte S.S. (2001). Procollagen II amino propeptide processing by ADAMTS-3. Insights on dermatosparaxis. J. Biol. Chem..

[57] Colige A., Ruggiero F., Vandenberghe I., Dubail J., Kesteloot F., Van Beeumen J., Beschin A., Brys L., Lapiere C.M., Nusgens B. (2005). Domains and maturation processes that regulate the activity of ADAMTS-2, a metalloproteinase cleaving the aminopropeptide of fibrillar procollagens types I-III and V. J. Biol. Chem..

[58] Vadon-Le Goff S., Kronenberg D., Bourhis J.M., Bijakowski C., Raynal N., Ruggiero F., Farndale R.W., Stocker W., Hulmes D.J., Moali C. (2011). Procollagen C-proteinase enhancer stimulates procollagen processing by binding to the C-propeptide region only. J. Biol. Chem..

[59] Hubmacher D., Wang L.W., Mecham R.P., Reinhardt D.P., Apte S.S. (2015). Adamtsl2 deletion results in bronchial fibrillin microfibril accumulation and bronchial epithelial dysplasia—A novel mouse model providing insights into geleophysic dysplasia. Dis. Model. Mech..

[60] Pingel J., Kampmann M.L., Andersen J.D., Wong C., Dossing S., Borsting C., Nielsen J.B. (2021). Gene expressions in cerebral palsy subjects reveal structural and functional changes in the gastrocnemius muscle that are closely associated with passive muscle stiffness. Cell Tissue Res..

[61] Smith L.R., Chambers H.G., Subramaniam S., Lieber R.L. (2012). Transcriptional abnormalities of hamstring muscle contractures in children with cerebral palsy. PLoS ONE.

[62] Smith L.R., Pontén E., Hedström Y., Ward S.R., Chambers H.G., Subramaniam S., Lieber R.L. (2009). Novel transcriptional profile in wrist muscles from cerebral palsy patients. BMC Med. Genom..

[63] Jones G.C., Corps A.N., Pennington C.J., Clark I.M., Edwards D.R., Bradley M.M., Hazleman B.L., Riley G.P. (2006). Expression profiling of metalloproteinases and tissue inhibitors of metalloproteinases in normal and degenerate human achilles tendon. Arthritis Rheum..

[64] Jelinsky S.A., Rodeo S.A., Li J., Gulotta L.V., Archambault J.M., Seeherman H.J. (2011). Regulation of gene expression in human tendinopathy. BMC Musculoskelet. Disord..

[65] Ireland D., Harrall R., Curry V., Holloway G., Hackney R., Hazleman B., Riley G. (2001). Multiple changes in gene expression in chronic human Achilles tendinopathy. Matrix Biol..

[66] Dean B.J., Franklin S.L., Carr A.J. (2012). A systematic review of the histological and molecular changes in rotator cuff disease. Bone Jt. Res..

[67] Arvind V., Huang A.H. (2021). Reparative and Maladaptive Inflammation in Tendon Healing. Front. Bioeng. Biotechnol..

[68] Ellis I., Schnabel L.V., Berglund A.K. (2022). Defining the Profile: Characterizing Cytokines in Tendon Injury to Improve Clinical Therapy. J. Immunol. Regen. Med..

[69] Jomaa G., Kwan C.K., Fu S.C., Ling S.K., Chan K.M., Yung P.S., Rolf C. (2020). A systematic review of inflammatory cells and markers in human tendinopathy. BMC Musculoskelet. Disord..

[70] Russo V., El Khatib M., Prencipe G., Citeroni M.R., Faydaver M., Mauro A., Berardinelli P., Cervero-Varona A., Haidar-Montes A.A., Turriani M. (2022). Tendon Immune Regeneration: Insights on the Synergetic Role of Stem and Immune Cells during Tendon Regeneration. Cells.

[71] Langberg H., Rosendal L., Kjaer M. (2001). Training-induced changes in peritendinous type I collagen turnover determined by microdialysis in humans. J. Physiol..

[72] Arampatzis A., Karamanidis K., Albracht K. (2007). Adaptational responses of the human Achilles tendon by modulation of the applied cyclic strain magnitude. J. Exp. Biol..

[73] Couppe C., Kongsgaard M., Aagaard P., Hansen P., Bojsen-Moller J., Kjaer M., Magnusson S.P. (2008). Habitual loading results in tendon hypertrophy and increased stiffness of the human patellar tendon. J. Appl. Physiol..

[74] Arampatzis A., Peper A., Bierbaum S., Albracht K. (2010). Plasticity of human Achilles tendon mechanical and morphological properties in response to cyclic strain. J. Biomech..

[75] Seynnes O.R., Erskine R.M., Maganaris C.N., Longo S., Simoneau E.M., Grosset J.F., Narici M.V. (2009). Training-induced changes in structural and mechanical properties of the patellar tendon are related to muscle hypertrophy but not to strength gains. J. Appl. Physiol..

[76] Bohm S., Mersmann F., Tettke M., Kraft M., Arampatzis A. (2014). Human Achilles tendon plasticity in response to cyclic strain: Effect of rate and duration. J. Exp. Biol..

[77] Bohm S., Mersmann F., Arampatzis A. (2015). Human tendon adaptation in response to mechanical loading: A systematic review and meta-analysis of exercise intervention studies on healthy adults. Sports Med. Open.

[78] Rosager S., Aagaard P., Dyhre-Poulsen P., Neergaard K., Kjaer M., Magnusson S.P. (2002). Load-displacement properties of the human triceps surae aponeurosis and tendon in runners and non-runners. Scand. J. Med. Sci. Sports.

[79] Sponbeck J.K., Perkins C.L., Berg M.J., Rigby J.H. (2017). Achilles Tendon Cross Sectional Area Changes Over a Division I NCAA Cross Country Season. Int. J. Exerc. Sci..

[80] Kongsgaard M., Reitelseder S., Pedersen T.G., Holm L., Aagaard P., Kjaer M., Magnusson S.P. (2007). Region specific patellar tendon hypertrophy in humans following resistance training. Acta Physiol..

