# Supplementary material for: Whole Genome Expression Profiling of Semitendinosus Tendons from Children with Diplegic and Tetraplegic Cerebral Palsy
Source: Biomedicines. 2023 Oct 28;11(11):2918. doi: 10.3390/biomedicines11112918 (PMC10669597; doi:10.3390/biomedicines11112918)
Supplement: Supplementary file 1 [file biomedicines-11-02918-s001.zip › Table S1.pdf]

Table S1. Oligos used for quantitative real-time PCR (qRT-PCR)

Primers	Sense	Reverse
COL1A1	GCATGTCTGGTTCGGCGAGA	ATGTTCTGGGAGGCCTCGGT
COL1A2	CCTAGCAACATGCCAATC	GACCATCTTCACCATCTCT
P4HA3	ACTGTTCCTATGAGACCAAT	CACCACTGACCTCTGTAG
KERA	CGCACAATCAACTCACAA	CATATTACAGAGACATTCACACT
SPARC	GCTGGATGAGAACAACAC	AAGAAGTGGCAGGAAGAG
TNMD	AGACAAGCAAGTGAGGAA	CGGCAGTAAATACAACAATAAC
MMP2	GCCAACTACAACTTCTTCC	GCATCATCCACTGTCTCT
GJA1	CACTTGAAGCAGATTGAGATAA	CTTGAAGAGGATACTGATGATG
CXCL10	CATCAGCATTAGTAATCAACCT	ACATCTCTTCTCACCCTTC
TP63	CAGGAAGAGACAGGAAGG	TGGTAAGTATAACAGTTCATCATC
GAPDH	TGACAACAGCCTCAAGAT	GTCCTTCCACGATACCAA
